# End user and forecaster interpretations of the European Avalanche Danger Scale: A study of avalanche probability judgments in Scotland

**DOI:** 10.1111/risa.70016

**Published:** 2025-03-11

**Authors:** Philip A. Ebert, David L. Miller, David A. Comerford, Mark Diggins

**Affiliations:** ^1^ Division of Law and Philosophy University of Stirling Stirling UK; ^2^ Biomathematics and Statistics Scotland Invergowrie UK; ^3^ UK Centre for Ecology & Hydrology Lancaster Environment Centre Lancaster UK; ^4^ Department of Economics University of Stirling Stirling UK; ^5^ Scottish Avalanche Information Service Aviemore UK

**Keywords:** avalanche risk communication, risk perception, verbal probability

## Abstract

We investigate Scottish end users' and professional forecasters' risk perception in relation to the 5‐point European Avalanche Danger Scale by eliciting numerical estimates of the probability of triggering an avalanche. Our main findings are that neither end users nor professional forecasters interpret the avalanche danger scale as intended, that is, in an exponential fashion. Second, we find that numerical interpretations by end users and professional forecasters have high variance, but are similar, in that both groups tend to overestimate the probability of triggering an avalanche and underestimate the relative risk increase. Finally, we find significant differences in the perceived probability of triggering an avalanche relative to a low or moderate avalanche danger level, and in the numerical interpretation of verbal probability terms depending on whether respondents provide their estimates using a frequency or a percentage chance format. We summarize our findings by identifying important lessons to improve avalanche risk understanding and its communication.

## 1 INTRODUCTION

In the European Alps and North America, there are around 140 avalanche fatalities each year (Peitzsch et al., 2020; Techel et al., 2016). Accidents usually involve skiers, mountaineers, and snow‐mobilers (Birkeland et al., 2017). Roughly, 90–95% of avalanche incidents with human involvement are human‐triggered avalanches—rather than natural avalanches—triggered usually by the victim(s) (Schweizer & Techel, 2017). Studies indicate that cognitive, social, and other biases play a role in avalanche incidents (Atkins, 2000; McCammon, 2004). As a result, avalanche research and education have taken a behavioral turn to investigate the cognitive and social factors in avalanche decision‐making (for an overview, see Hetland et al., 2025). Our contribution focuses on how end users and professional avalanche forecasters interpret the avalanche danger scale and the verbal probability terms used in avalanche forecasts.

**FIGURE 1:**
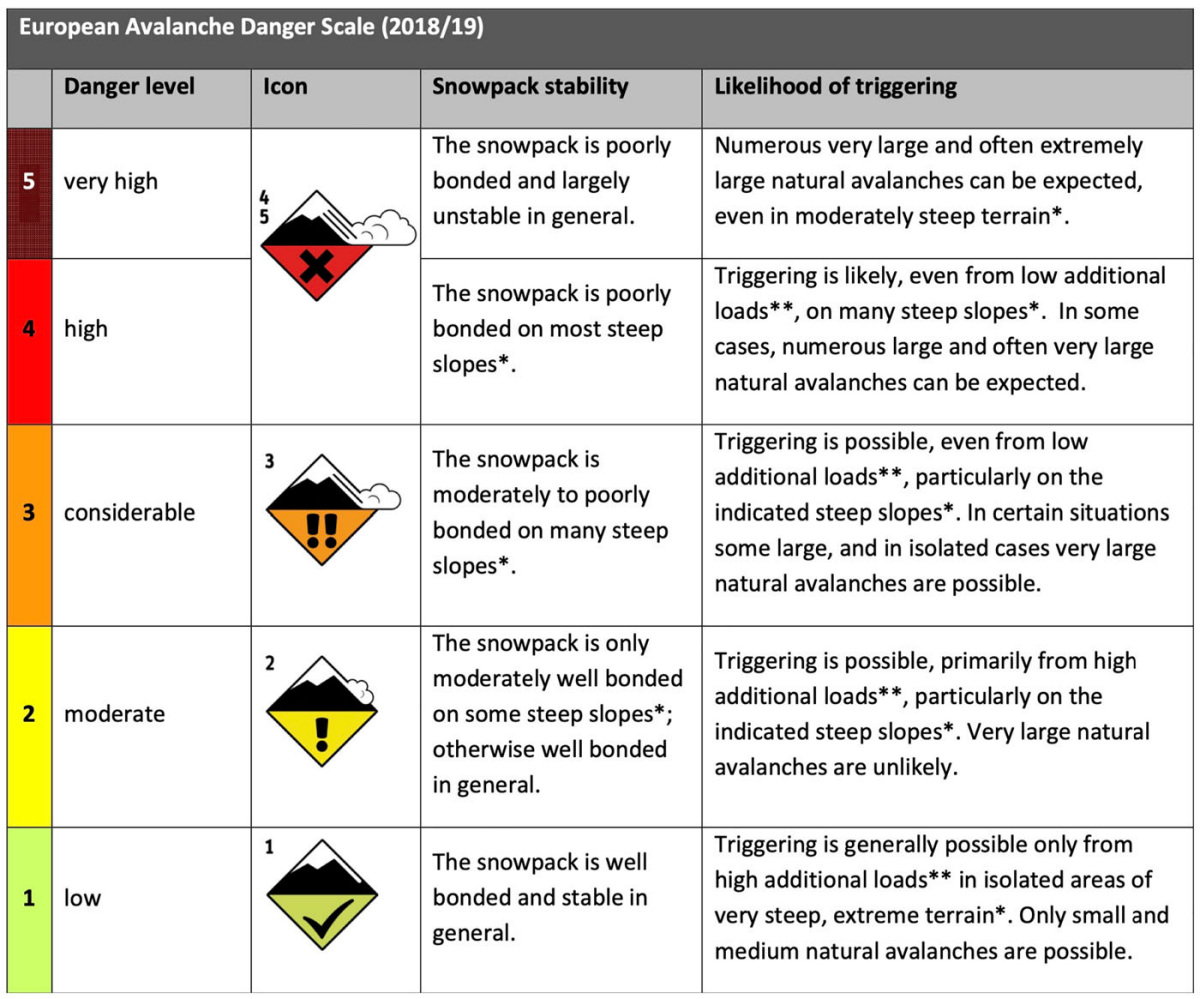
The EAWS Avalanche Danger Scale including snowpack stability and likelihood of triggering descriptors. (*) indicated steep slopes are more than 30 degrees, moderately steep terrain are slopes shallower than 30 degrees; (**) low additional loads denotes an individual skier, while high additional loads indicates two or more skiers.

Avalanche forecasts are usually issued by regional or national avalanche warning services. All European avalanche services use the EAWS standardized color‐coded 5‐point ordinal *avalanche danger scale* that ranges from *low* to *very high*, see Figure 1 (EAWS, 2023a).

The avalanche danger level is a function of three main determinants: snowpack stability (inversely related to the probability of an avalanche triggering), the frequency distribution of snowpack stability (how widespread trigger points with the lowest snowpack stability rating are), and the expected avalanche size. The resulting avalanche danger level applies to a region of at least 50–100 km^2^. Forecasters use the EAWS Matrix, a decision‐tool that combines the three determinants, to forecast danger levels in a consistent way (EAWS, 2023b).

**FIGURE 2:**
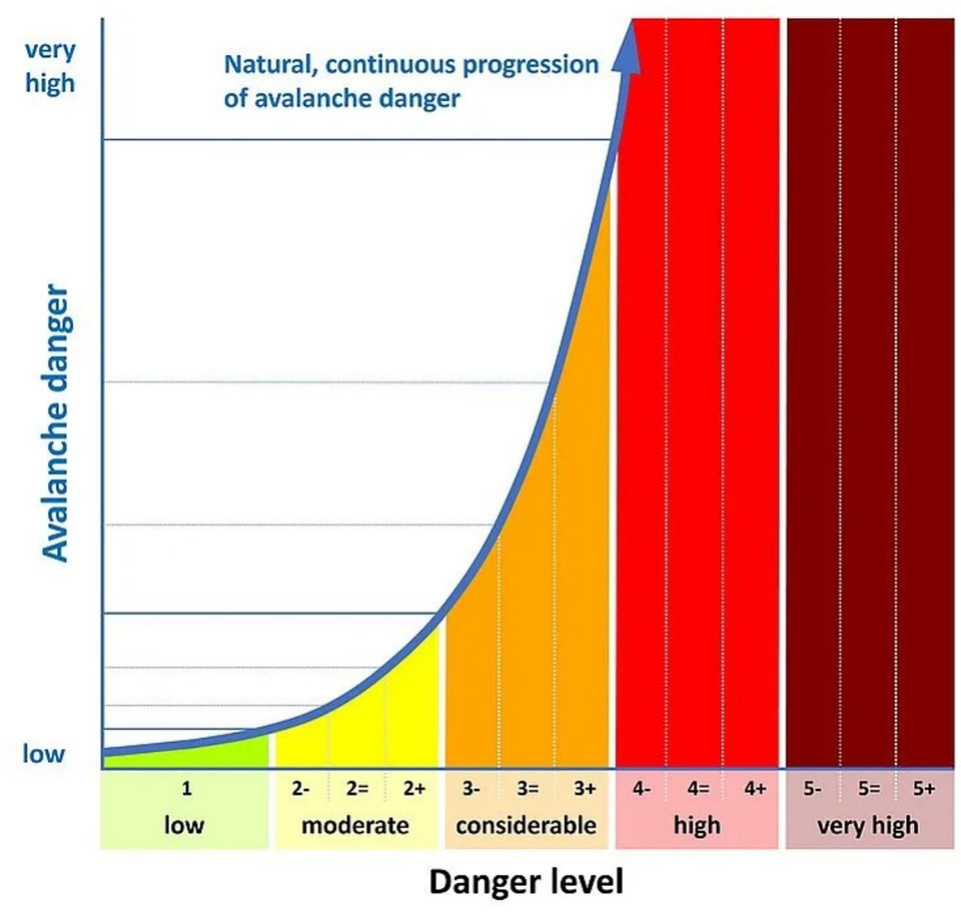
The exponential and continuous increase of the avalanche danger in relation to the reported avalanche danger levels as presented by the Swiss Forecasting Service (SLF, 2022). Note that sublevels are currently only in use in Switzerland and have not been adopted by the EAWS.

Each EAWS avalanche danger level has descriptors that specify the avalanche conditions for that level in relation to “snowpack stability” and the “likelihood of triggering” an avalanche (Figure 1). The latter descriptor includes information about the expected *trigger likelihood* presented using verbal probabilities (“possible,” “likely”). One aspect of the avalanche danger scale is that scientists interpret the avalanche danger to increase *exponentially* (Schweizer et al., 2020). The official guidance by EAWS highlights the exponential nature of the increase of avalanche danger (EAWS, 2023a), similar to Figure 2. Furthermore, the Swiss Avalanche Service notes that: “the probability of an avalanche triggering increases sharply as the danger level rises”(SLF, 2022).

There has been some research focusing on end user understanding and interpretation of the avalanche danger levels. Engeset et al. (2018) provide the first large‐scale study of end users of avalanche forecasts identifying how different modes of communication affect their level of comprehension. Using a mixed methods approach, St. Clair et al. (2021) have identified different user groups of avalanche forecast who each draw on different levels of specificity in their avalanche risk assessment. Terum et al. (2023) identified the effect of increasing and decreasing trend in danger ratings on end users' perception of the current danger rating level. Finally, the study by Morgan et al. (2023) found that roughly 65% of North American end users increased their numerical interpretation of the North American avalanche danger scale—a scale that is modeled on the EAWS avalanche scale and also uses five levels (Statham et al., 2010)—in a linear fashion.

The novel contribution of our research is to focus on end users' and professional avalanche forecasters' *probabilistic* interpretation of the avalanche danger levels and of the verbal probability terms employed in avalanche forecasts. This numerical perspective has received renewed interest for avalanche risk communication for three reasons. First, the EAWS has introduced the use of numerical estimates in the EAWS Matrix to ensure a consistent forecasting approach. Second, a numerical approach provides objectively testable verification procedures for the avalanche danger levels. Third, recent research suggests that receivers of risk information prefer numerical estimates but that senders are more hesitant to use them: a phenomenon called the *communication mode preference paradox* (Dhami & Mandel, 2022; Irwin & Mandel, 2023).

There are good reasons specific to avalanche risk communication to avoid numerical estimates. First, there is the issue of *scope* (Ebert & Milne, 2022, p.550‐51): avalanche danger levels apply in the first instance to a wider region and not to individual slopes where we can expect be a high degree of variability. Second, human behavior and decision‐making on a particular slope does affect the probability of triggering an avalanche, so the relevant estimates are not independent of the individual's behavior. Third, we are dealing with rare (when considering individual slopes) and severe events where it is often difficult to interpret the relevant numerical probabilities: given the stakes involved, it might be misleading, or worse, irresponsible, to interpret a 10% chance of triggering an avalanche on a given slope as “unlikely,” as standard usage of the term would suggest. Some professional avalanche forecasters have struggled with similar issues when explaining why numerical estimates are usually avoided in operational contexts (Statham et al., 2018, p. 682).

The aim of this contribution is, first, to gain a better understanding of how *end users* and *forecasters* interpret one aspect of the avalanche danger scale—the probability of avalanche triggering aspect—using numerical estimates. In Section 2, we investigate whether end users and forecasters interpret the increase of the probability of triggering an avalanche given an increase in avalanche danger levels as intended, that is, exponentially (“sharply”). The second aim, discussed in Section 3, is to investigate how end users and avalanche professionals numerically interpret the trigger likelihood terms that are used in the descriptors of the avalanche danger level. Our third aim, addressed in both studies, is to ensure robustness of our estimates by eliciting end users' estimates using different response formats. To that end, we distinguish between frequency and percentage chance formats and test whether there are within and between response format variations.

## 2 EXPONENTIAL VERSUS LINEAR INTERPRETATION OF THE AVALANCHE DANGER SCALE

We first present our survey of end users of the Scottish Avalanche Information Service (SAIS), followed by a shorter survey of professional avalanche forecasters of the SAIS.

### 2.1 End user's numerical interpretation of the danger scale

Our study builds on an earlier study by Morgan et al. (2023), which used the North American avalanche scale and a North American sample population. Soliciting responses for perceived avalanche danger *per se* (described using the three determinants) on a 0–100 scale, they found that perceived avalanche danger increases in a linear fashion for most respondents. By soliciting responses for perceived avalanche danger, their approach directly assesses end users interpretation of the danger scale. Instead, our study focuses on the probability aspect of avalanche danger, which allows for the standard interpretation of end user responses using probability and frequency scales and for well‐defined comparisons between individuals.

#### 2.1.1 Survey participants

The survey was presented as a collaboration between the University of Stirling and the SAIS. Participants were recruited with the help of the SAIS website, word of mouth, and online advertisements. The survey received Ethics approval (GUEP(2021) 1012) from the University of Stirling. Data and R code are here: https://osf.io/3b78y/

The survey was designed using Qualtrics and open from March 2021 to April 2021. 678 of 1193 respondents completed it and reported prior acquaintance with the SAIS avalanche forecasts. Participants were not required to answer any questions, which resulted in variations in sample sizes throughout. The median age category was 45–54 (per‐age‐category counts are: 18–24: 36, 25–34: 110, 35–44: 144, 45–54: 161, 55–64: 147, 65–74: 66, 75–84: 7). 543 respondents identified as male, 117 as female, and one selected neither. Level of formal education was skewed toward higher education (postgraduate: 293, undergraduate: 330, A‐level/high school: 93, never finished school: 3).

When asked about their experience in winter sports, the modal experience level was more than 20 years, with 535 respondents having more than 5 years experience (1–2 winters: 38, 3–5 winters: 95, 6–10 winters: 106, 11–20 winters: 166, more than 20 winters: 266). Regarding the use of forecasts, 60 respondents reported to only use the avalanche danger scale, 197 reported to also use altitude and aspect information, 401 reported to also use information about the type of avalanche predicted (“avalanche problems”).

#### 2.1.2 Experimental questions and design

We explore end users' risk perception by soliciting numerical estimates of the likelihood of triggering an avalanche for a given avalanche danger level. Respondents were asked to provide estimates for all five avalanche danger levels in the same order. We randomly assigned respondents to one of three response formats. The *percentage chance* format is widely used in state‐of‐the‐art administrative surveys such as the Survey of Consumer Expectations (Bruine de Bruin & Fischhoff, 2017). The *frequency* format is used by European forecasters in operational contexts, and research suggests that a frequency format provides a more accurate measure of respondents' probabilistic beliefs (e.g., Bell et al., 2020; Comerford & Robinson, 2017) and that lay people can better articulate their probabalistic beliefs in a frequency format (Comerford, 2019; Gigerenzer & Hoffrage, 1995).

We used a percentage chance format and two frequency formats (x‐many avalanche per‐100 *slopes* and x‐many avalanches per 100‐*days*). Respondents were asked to provide their *best estimate* of the likelihood of an avalanche for each avalanche danger level in ascending order from *low* to *very high* using a sliding numerical scale (0–100) allowing for whole number responses only. The three response formats were:

*Percentage chance*
  Imagine a large steep snow slope in an area that is rated to have one of the five hazard levels. Please provide your best ebstimate of the percentage chance that an avalanche will be triggered by a person crossing it for each hazard levels.
*Frequency slope (days)*
  Imagine a large steep snow slope in an area that is rated to have one of the five hazard levels. Out of a 100 slopes with the same characteristics and hazard level (*100 days at the given hazard level*), how many slopes will avalanche (*how often will an avalanche be triggered*) if a person crosses it? Please provide your best estimate for each hazard level.

#### 2.1.3 Statistical methods

Respondents' answer was modeled as a continuous response. Following Benjamin et al. (2018), we used α=0.005 throughout, interpreting p‐values between 0.005 and 0.05 as providing *suggestive* evidence.

To assess how well participants' responses match the assumed exponential (“sharp”) increase in the probability of triggering an avalanche, we used three methods. First, we fitted a generalized additive mixed model (GAMM; e.g., Wood, 2017) to the 0–100 responses, with danger level as predictor. To assess whether the relationship was exponential, we fitted a model where the danger level was a linear effect, plus a smooth (spline) effect for danger level. We allowed for differing shapes per response format by duplicating the smooth for each format (using model “I” of Pedersen et al., 2019). We used a random effect for each individual accounting for variations from other variables (gender, age, etc.). Using this method, we assess whether the responses are best modeled using a linear function or whether there are “kinks” in the regression, suggesting a nonlinear response pattern.

Second, using both the mean and median responses, we investigate the *factor* by which the perceived probability of triggering an avalanche increases with each danger level. An exponential interpretation is formally characterized by a constant factor.

Since it is unclear whether forecasters have the formal definition of exponentiality in mind, we also assessed responses using a less demanding definition that captures the idea of a “sharp” increase. The third method regards an exponential increase as one where the value by which the probability of avalanche triggering increases is itself increasing for each danger level (see Table 1 for a precise characterization).

**TABLE 1:** Third criterion for exponentiality: UR(x) is the user response at a given avalanche danger level x, where 1 is danger level *Low*, 2 is *Moderate*, 3 is *Considerable*, 4 is *High*, and 5 is *Very High*.

Condition	Requirement
Condition 1:	UR(2) − UR(1) > 0
Condition 2:	UR(3) − UR(2) > UR(2) − UR(1)
Condition 3:	UR(4) − UR(3) > UR(3) − UR(2)
Condition 4:	UR(5) − UR(4) > UR(4) − UR(3)

For example, users who associate 5 with *low* and 10 with *moderate*, exhibit an exponential increase only if their response to *considerable* is 16 or greater. We applied this definition to individual responses separating them into three groups: a response that meets all four conditions in Table 1 is an *exponential* one. Since in Scotland the highest avalanche danger level is never used, we also included an assessment that considers only the first four levels, that is, we regard a response that meets the first three conditions as a *limited exponential* one. All others are considered *nonexponential*.

We also investigated whether there are differences in end user estimates relative to the different formats. We used three hypothesis tests for this purpose: first, we applied Levene's test to assess whether variances were different between the response formats at each avalanche danger level (Levene, 1960). The Kruskal–Wallis test was then used to determine if the responses came from the same distribution, that is, whether there are any differences in responses within danger level between the response formats (Kruskal & Wallis, 1952). If a difference was detected, we used Dunn's test with a Bonferroni correction for multiple testing (Dunn, 1964) to ascertain which formats were significantly different from each other.

We calculated effect sizes for each hypothesis test, following Tomczak and Tomczak (2014): for the Levene's test, we report the standard deviations and sample sizes, for the Kruskal–Wallis test, we reported both the $\eta^{2}$ metric (providing a proportion of variance explained by the group) and $E^{2}$ (varying between 0 and 1, from none to full dependence). For the Dunn's test, we report the $r^{2}$ statistic (correlation coefficient).

#### 2.1.4 Results

We recorded 1596 danger level evaluations (responses from 320 individuals across five questions, with one noncomplete evaluation). We discarded one response (giving a total of 1595) where the response was 0 for a non‐*low* danger level (0 was the default value on the scale). Throughout our analysis, we excluded six end user responses that were not even weakly monotonic (two of which even strictly decreasing). We interpret such a response pattern as indicative of a basic misunderstanding of the nature of the avalanche danger scale (this exclusion made no substantial difference to our results).

**FIGURE 3:**
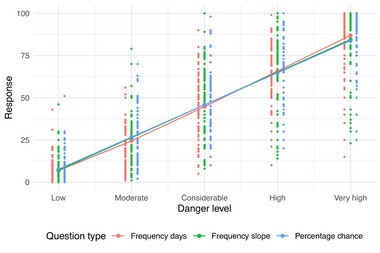
Relationship between danger level and the end user responses. Lines and large dots along the lines show the fitted relationship from a generalized additive model using the danger level linear plus smooth effect: the linear effect clearly dominates here. Small dots show the data, with a horizontal dodge applied in the plot to allow all points to be seen. Note that the frequency slope line is nearly entirely covered by the percentage chance line.

The resulting model showed that the relationship was almost linear. The linear term was significant ($p<1\times 10^{-16}$). The smooth terms (one per response format) were not significantly different from zero (p= 0.0872, 0.4435, 0.6788 for frequency days, frequency slopes, percentage chance, respectively). As the smooth terms are extremely flexible, we would expect them to fit to any deviation from linearity, but they did not. It, therefore, appears that the average end user interpretation of the scale is linear. Figure 3 shows the fitted relationship, which appears linear with no “kinks” in the regression line.

**FIGURE 4:**
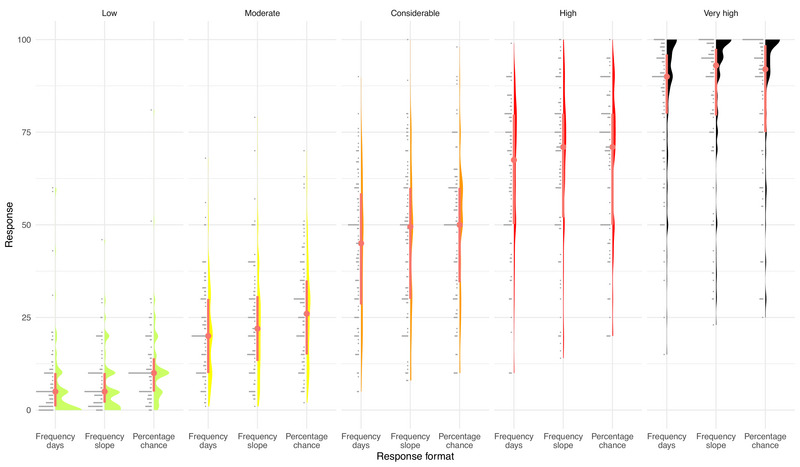
Raincloud plot showing the responses of survey participants when asked to assess the likelihood of avalanche for each danger level (low, moderate, considerable, high, and very high; top of plot) as response format (frequency days, frequency slopes, percentage chance; bottom of plot) varies. For each subplot, the “rain” (horizontal lines to the left) gives the raw data histogram, the “cloud” itself is a kernel density smooth of that data. The median is given by the dot, with the thick line indicating the interquartile range of the data.

Figure 4 presents a raincloud plot showing the distribution of responses for each danger level and response format. Responses for *low* and *very high* tended to be located at the lower and upper end of the response scale. *Considerable*, *high*, and *very high* have a higher variance than the other ratings (see Table A1). We find that the factor by which the perceived probability of triggering an avalanche increases with each danger level is strictly decreasing. Looking at the average responses (independent of response format), the factor by which the probability of triggering an avalanche increases from the danger levels *low* (mean: 8.66; median: 5) to *moderate* (23.9; 22) is 2.76 (mean) and 4.4 (median), from *moderate* to *considerable* (46.1; 50), it is 1.93 and 2.3, from *considerable* to *high* (65.9; 70), it is 1.43 and 1.4, and from *high* to *very high* (83.9; 90), it is 1.27 and 1.3. This provides further evidence that end user interpretation of the probability of triggering an avalanche is not increasing exponentially since this factor should be constant on an exponential interpretation.

**TABLE 2:** Proportion of respondents fulfilling the minimal condition of expontentiality independent of response format (“Total”), and relative to response format.

Condition	Total	${\mathrm{Frequency}}_{days}$	${\mathrm{Frequency}}_{slopes}$	Percentage
Condition 1:	98.75%	98.11%	99.07%	99.07%
Condition 2:	70.62%	69.81%	73.83%	68.22%
Condition 3:	25.31%	23.58%	29.99%	22.42%
Condition 4:	10.63%	9.433%	14.02%	8.41%

**TABLE 3:** Dunn's test p‐values, sample sizes (n), and effect sizes ($r^{2}$) for differences between response formats, per danger level.

Danger level	Comparison	Dunn's *p*‐value	*n*	$r^{2}$
Low	Slopes‐Percentage	0.0005506	214	0.0593128
	Days‐Percentage	0.0000013	212	0.1140437
	Days‐Slopes	0.2554845	212	0.0088684
Moderate	Slopes‐Percentage	0.0944772	213	0.0162300
	Days‐Percentage	0.0022835	213	0.0471895
	Days‐Slopes	0.2862990	212	0.0080704

The two frequency formats are referred to by their denominator “Slopes” and “Days,” respectively, while the percentage chance format is referred to using “Percentage.”

**TABLE 4:** Comparing the numerical estimates of probability terms relative to the different response formats.

Probability	Comparison	*p*‐value	*n*	$r^{2}$
Possible	Frequency‐Percentage chance	$<10^{-5}$	218	0.07
	Certainty‐Frequency	$<10^{-16}$	229	0.16
	Certainty‐Percentage	0.08	223	0.02
Likely	Frequency‐Percentage chance	$<10^{-6}$	218	0.12
	Certainty‐Frequency	$<10^{-16}$	229	0.19
	Certainty‐Percentage chance	0.23	223	0.01
Very likely	Frequency‐Percentage chance	$<10^{-3}$	218	0.07
	Certainty‐Frequency	$<10^{-16}$	229	0.15
	Certainty‐Percentage chance	0.11	223	0.01

We note p‐values for the Dunn's test between formats and probability terms, and effect size.

**FIGURE 5:**
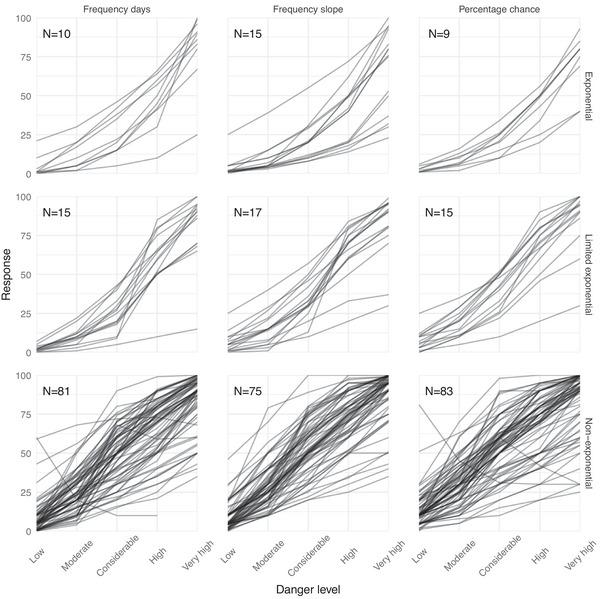
End user responses estimating the probability of an avalanche at each avalanche danger level (faceted by response format). Each line is the response from one user. Columns give the response formats and rows show responses that are exponential (conditions 1–4 met), limited exponential (conditions 1–3 met), and nonexponential (remaining). We included all responses in this figure, that is, also those that were excluded from the analysis.

Looking at the individual responses (see Figure 5) and our third condition of exponentiality (Table 1), we find that independent of response format, 98.75% respondents meet Condition 1, 70.62% meet Condition 2, then there is a significant drop, and only 25.31% meet Condition 3, while just 10.63% meet Condition 4 and thus offer an exponential response pattern (see Table 2). Based on Figure 5, we can also note that the linear fit observed in Figure 3 is broadly representative and not due to possible averaging effects with users exhibiting either a convex or a concave response pattern. In summary, there is robust evidence that a substantial majority of end users fail to interpret the probability of triggering an avalanche as intended.

In order to address our third aim, we performed the following tests to ascertain whether there are within and between response format variations: Levene's test for the homogeneity of the variance between the response format for each level did not yield significant differences (p= 0.6412, 0.3417, 0.6253, 0.9706, and 0.7797; see Table A1 for effect sizes). The Kruskal–Wallis test showed that for the *low* danger level there were significant differences between the response formats ($p=2.458\times 10^{-6}$) and for the *moderate* danger level, we found suggestive evidence of a difference (p=0.00623). The follow‐up Dunn's test showed that at *low* danger level there was a significant difference between the frequency days and percentage formats ($p<1.3\times 10^{-6}$) and the frequency slope and percentage formats (p=0.0006). For the *moderate* danger level, frequency days and percentage chance formats were significantly different (p=0.0023), but we found no statistically significant difference between frequency slope and percentage chance (p=0.0945). The effect sizes for the Kruskal–Wallis tests are shown in Table A2, for the Dunn's tests, see Table 3.

### 2.2 Professional avalanche forecaster's numerical interpretation of the danger scale

#### 2.2.1 Survey participants

Participants were active avalanche forecasters employed by the SAIS. Out of a total 24 active professional forecasters, we received 19 valid responses. In order to guarantee their anonymity, we did not ask for any generic information such as gender, age, and experience in avalanche forecasting. All avalanche forecasters have received forecasting‐specific professional training and undergo continual professional development. Additionally, most forecasters are mountain professionals with outdoor leadership qualifications. Their forecasting experiences range from a minimum of 5 years to 20 or more years.

#### 2.2.2 Experimental questions and design

We used the same question as previously. Given the low expected sample size, we used only on one response format, namely, the frequency‐slopes format.

#### 2.2.3 Statistical methods

Given the low sample size, we did not fit a GAMM to the data, but we did apply the other two criteria of exponentiality as outlined in Section 2.1.3. We used a linear regression model to show the general trend of the data when plotting the comparisons between users and forecasters.

#### 2.2.4 Results

**FIGURE 6:**
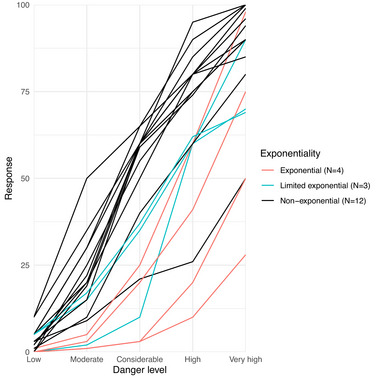
Forecasters' responses estimating the probability of an avalanche at each avalanche danger level. Each line is the response from one forecaster. Blue lines are limited exponential responses, while red lines are exponential responses.

Plotting the individual forecaster responses in Figure 6, we can identify a range of response types including concave, linear, convex, and s‐shaped response‐patterns. We find that avalanche forecasters fared better with respect to the third criterion of exponentiality (Table 1): 100% of avalanche forecasters meet Condition 1, 89.47% meet Condition 2, again there is a significant drop given that only 36.84% (more than one in three compared to one in four for end users) meet Condition 3 and offer a limited exponential response, while just 21.63% (roughly, one in five compared to one in 10 for end users) meet Condition 4 and count as offering an exponential interpretation.

**FIGURE 7:**
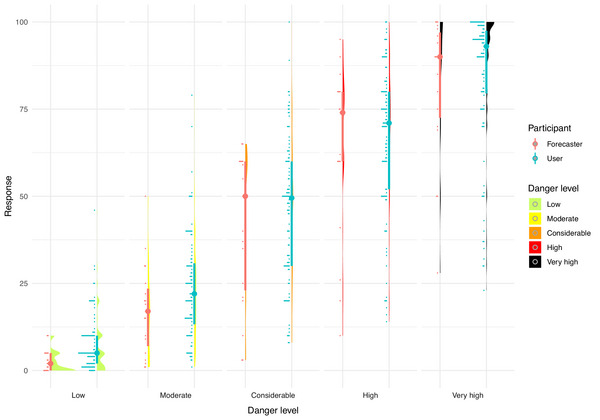
Raincloud plot showing the responses of forecasters when asked to assess the likelihood of avalanche for each danger level (low, moderate, considerable, high, and very high; horizontal axis). Corresponding answers for users (using the same frequency format only) are provided in blue for comparison. For each danger level, the “rain” (horizontal lines to the left) gives the raw data histogram, the “cloud” itself is a kernel density smooth of that data. The median is given by the dot, with the thick line indicating the interquartile range of the data.

We also visually compare end users and forecaster responses and find that the two groups provide broadly similar estimates (Figure 7). Looking at the mean and median responses, we find that the factor by which the probability of triggering an avalanche increases from danger level *low* (3; 2) to *moderate* (17.3; 17) is 5.77 (mean) and 8.5 (median), from *moderate* to *considerable* (41.5; 50), it is 2.4 and 2.94, from *considerable* to *high* (63.8; 74), it is 1.54 and 1.48, and from *high* to *very high* (81.8; 90), it is 1.28 and 1.2. The main difference between end users and forecasters on this measure is that the latter tend to provide lower average scores for the danger levels *low* and *moderate*, leading to higher factors at the lower danger levels compared to end users. But just as with end users, the factor itself is strictly decreasing with increasing danger level.

### 2.3 Discussion

#### 2.3.1 Linear interpretation of the avalanche danger scale

We found clear evidence that end users and forecasters in Scotland interpret the increase in probability of an avalanche triggering as the danger level increases in a linear manner. As displayed in Figure 3, the average user response does not exhibit a classic exponential response patterns but rather it is best modeled using linear regression.

Also, using the formal interpretation of exponentiality, we found that the factor by which the probability of avalanche triggering increases for an increase in avalanche danger level is *strictly decreasing* rather than being constant. This provides further evidence that the end user interpretation of the danger scale is not exponential and does not exhibit a “sharp” increase.

Finally, looking at individual responses, and adopting a less demanding interpretation of exponentiality (Table 1), we find that roughly 90% of end users and 80% of forecasters do not exhibit an exponential interpretation, while roughly 75% of end users and 65% of forecasters are not even showing a limited exponential interpretation. This finding was robust across the three response formats. As such, the intended interpretation that “the probability of an avalanche triggering increases *sharply* as the danger level rises”(SLF, 2022) is not widely shared by end users nor by forecasters.

Our results have clear implications for avalanche risk communication since they suggest that end users tend to think that the factor by which the probability of avalanche triggering increases from *low* to *moderate* is greater than from *moderate* to *considerable* (see Section 2.1.4). However, no such decrease is found in studies that have attempted to estimate the relevant factor. Jamieson et al. (2009) suggest, using a exploratory risk analysis and a simplified event tree, that there is roughly a 10‐fold increase in triggering probability when the regional danger increases by one level throughout. In contrast, using GPS and accident data, Winkler et al. (2021) found that the observed personal avalanche risk in Switzerland increases roughly by factor 4 from low to moderate, as well as from moderate to considerable. The latter study is closer to earlier studies that also found a constant factor of2 or 3 (Pfeifer, 2009; Techel et al., 2015). Our finding that the factor decreases suggests that end users, as well as professional forecasters, underestimate the relative avalanche risk increase when moving to higher danger levels. See, however, Section 2.3.4 for possible limitations interpreting this result.

#### 2.3.2 Comparing professional avalanche forecaster and end user interpretation of the avalanche danger

Even among professional avalanche forecasters, we found large variance in the responses and no clear peaks for all but the *low* avalanche danger level. In comparison with end users, forecasters tended to provide lower average responses for the two lowest danger levels, and a higher proportion of forecasters exhibited limited exponential and exponential response pattern.

The reasons for why even professional forecasters exhibit high variance, aside from low sample size, might be that forecasters associate a wide range of *best estimate* responses to each danger level given the spatial variability of the Scottish snowpack. Also, wide uncertainty ranges associated with making slope‐specific assessments can result in a wide range of judgments.

Finally, it is worth noting that neither end users nor professional forecasters are very well calibrated. Jamieson et al. (2009) suggest that 1% is a reasonable (upper limit) estimate of triggering a fresh slope on a *considerable* danger level without mitigation techniques. Similarly, Winkler et al. (2016) find that there are roughly 10 fatalities per 1 million ski‐touring days. If we assume, conservatively, that on each day only one avalanche slope is crossed, and that for each avalanche fatality, we have roughly 100 triggered avalanches, we arrive at a 1 in 1000 chance or 0.1% of triggering an avalanche per slope independent of avalanche danger level.

These considerations point toward limitations of our study, as well as possible improvements that should be made in future research which we discuss in Section 2.3.4. Despite this, the failure of most respondents to adopt an expontential interpretation and their miscalibration in relation to more realistic estimates raises important questions for avalanche risk communication—an issue we return to in Section 4.

#### 2.3.3 Response formats and variance

The study also provides interesting results to address our third aim, that is, whether the response format plays a role in end user interpretation. We found that the frequency response format leads in some cases to lower mean average scores compared to the percentage chance response format. One research hypothesis is that in a frequency format, individuals are considering first the event (avalanche) and then consider how often per 100 slopes/days it occurs. Extrapolating a rare event over the denominator may lead individuals to come up with a lower interpretation compared to providing a more direct percentage chance estimate of an avalanche on a given slope. Having said this, the fact that different denominators in a frequency format (slopes vs. days) showed no significantly different responses suggests that the frequency response format is robust across different denominators.

#### 2.3.4 Limitations of Study 1

There are three limitations to highlight that we plan to address in future studies. First, we used a slider scale to solicit responses which only allowed integer responses. A slider scale, especially on mobile devices, might make it more difficult for respondents to provide a low response (close to 1). Having said this, given that median responses for the avalanche dangers levels were 5, 22, 50, 70, and 90, for end users, it does not appear that the slider scale will have biased the answers substantially (except maybe for the low avalanche danger). Still, in future studies, we will drop the use of a slider in favor of an open text entry.

Second, our response format made it impossible to make estimates below the 1% range, which made it more difficult to provide realistic estimates. Having said this, it is worth noting that most estimates even by professional forecasters did not tend to be located at the bottom end of the scale. Still, in future studies, we will investigate how robust these estimates are using different response format (1 in 100 vs. 1 in 1000 or 10,000). Relatedly, not allowing sub‐1% responses made it also more difficult to meet the second formal definition of exponentiality, especially because we asked individuals to provide estimates for all five danger levels. Given this setup, a constant factor of 2 requires that the estimate for the low danger level is 6 or below, while a constant factor of 3 is only possible for a low danger level rating of 1. By adopting a wider response format and by soliciting fewer estimates, we can address these limitations in future studies.

Finally, this study focused on the perceived probability of triggering an avalanche. However, since the probability aspect is only one determinant of the avalanche danger scale, any inference about respondents' interpretation of the avalanche danger level *per se* has to be treated with care. Still, on the assumption that there are not many end users who will interpret the avalanche danger itself to increase exponentially despite interpreting the probability of avalanches to not increase in that manner, we can conclude that our findings provide further, albeit indirect, evidence for 2023Morgan et al.'s original finding that most end users do not adopt an exponential interpretation of the avalanche danger scale.

## 3 NUMERICAL INTERPRETATION OF VERBAL PROBABILITY TERMS IN AN AVALANCHE CONTEXT

Studies have shown that numerical interpretation of the probability terms such as “possible,” “likely” is associated with a range of numerical estimates (Vogel et al., 2022). For example, the term “likely” is standardly associated with a numerical estimate above 50% and in the 60–80% range (Teigen et al., 2022). However, numerous studies have shown that the relevant context can have a significant effect on how the terms are interpreted (Budescu & Wallsten, 1995). The aim of the second study is to investigate whether end users and professional forecasters are sensitive to differences in the trigger likelihood terms, how they interpret these terms numerically in an avalanche context, and to what extent the response format has an effect on their numerical estimates. As before, we first present the results of our study of end users followed by professional forecasters.

### 3.1 End user's numerical interpretation of verbal probability terms

#### 3.1.1 Survey participants

The same participants as in Section 2.1.1.

#### 3.1.2 Experimental questions and design

Study participants were first reminded that avalanche forecasts use a 5‐ point danger scale and then asked to provide numerical estimates for verbal probability terms once primed with the following introduction:

*Interpreting likelihood terms*
  Verbal descriptors like “possible,” “likely,” “very likely” are used to indicate the frequency of an avalanche on a given slope. When you read these terms in an avalanche forecast what do these terms mean to you?

Respondents were asked to offer a single point numerical estimates of triggering an avalanche for: “triggering is possible,” “triggering is likely,” and “triggering is very likely,” in the same order using a slider scale from 0 to 100 allowing for whole numbers only. Respondents were randomly assigned to one of three response formats which was independent of the randomization in Study 1. We used a percentage chance format, a frequency format, and a certainty format that asked people about their *degree of certainty* of triggering an avalanche. For the latter format only, we also added a descriptor for the mid‐point.

*Percentage chance*
  Please provide your best estimate of the percentage chance that an avalanche occurs, if triggering is possible/likely/very likely.
*Certainty*
  Using a scale from 0 (certain that no avalanche) to 100 (certain that there will be an avalanche), with the mid‐point at 50 (toss‐up), please indicate your confidence that an avalanche occurs, if […].
*Frequency*
  Please provide your best estimate of how many avalanches there will be out of 100 slopes, if […].

#### 3.1.3 Statistical methods

We used the Kruskal–Wallis and Dunn's tests to determine differences between verbal probability terms and between response formats for each verbal probability term. We reported effect sizes as described previously.

#### 3.1.4 Results

**FIGURE 8:**
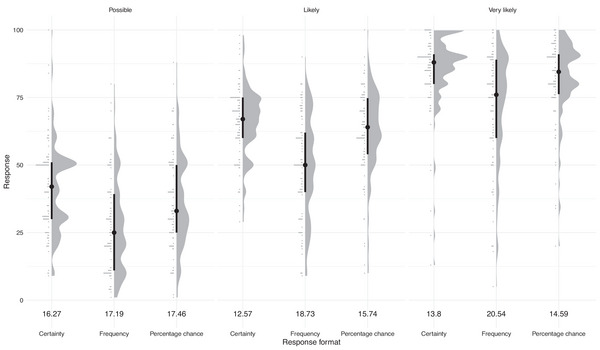
Raincloud plot showing the responses of end users when asked to provide a numerical estimate of verbal probability terms (possible, likely, very likely; top of plot) as the response format (frequency days, frequency slopes, percentage chance; bottom of plot) varies. For each subplot, the “rain” (horizontal lines to the left) gives the raw data histogram, the “cloud” itself is a kernel density smooth of that data. The median is given by the dot, with the thick line indicating the interquartile range of the data. Numbers under each plot give the standard deviation of that data.

A Kruskal–Wallis test showed that respondents provide significantly different estimates for “possible,” “likely,” and “very likely” (p‐value $=<2.2\times 10^{-16}$). Mean estimates, ignoring response format, for these terms are 35.4, 59.4, and 79.4, respectively. Figure 8 uses a raincloud plot to show the numerical estimates and the standard deviations for each term and response format combination. The range of estimates encompasses almost the whole scale.

We also tested for differences in end user estimates between response format. Post‐processing, we had 1005 responses, with 335 for each verbal probability term; sample sizes were approximately equal over the three response formats: certainty 351, frequency 336, and percentage chance 318. Using the Levene test, we found no significant evidence for heteroscedasticity between response formats for “possible” (p=0.8526) but there was a significant difference between response formats for “likely” and “very likely” (p=0.0001081 and 2.006$\times 10^{-5}$, respectively).

The Kruskal–Wallis test was significant for all verbal probability terms, indicating a difference between response formats ($p=4.642\times 10^{-9}$, $3.053\times 10^{-11}$ and $1.499\times 10^{-8}$, in ascending order, as per Figure 8). Following this, we performed Dunn's test to determine which response formats were significantly different. In all cases, the frequency versus certainty and frequency versus percentage chance were significantly different, whereas percentage chance versus certainty were not significantly different. These results are summarized in Table 4. Effect sizes are reported in Figure 8 and Tables 4 and A3.

Differences in estimates given different response formats are consistent with our finding in Study 1 in that the frequency format leads to significantly lower numerical estimates.

### 3.2 Professional avalanche forecaster's numerical interpretation of verbal probability terms

#### 3.2.1 Survey participants

Same as in Study 2.2.

#### 3.2.2 Experimental questions and design

We used the same experimental design and questions for professional avalanche forecasters. Given the low expected sample size, we used only one response format, namely, the frequency format.

#### 3.2.3 Statistical methods

Given the low sample size, we did not apply any significance tests to our results. But we present professional forecaster responses for each term including a basic analysis (means and standard deviation).

#### 3.2.4 Results

**FIGURE 9:**
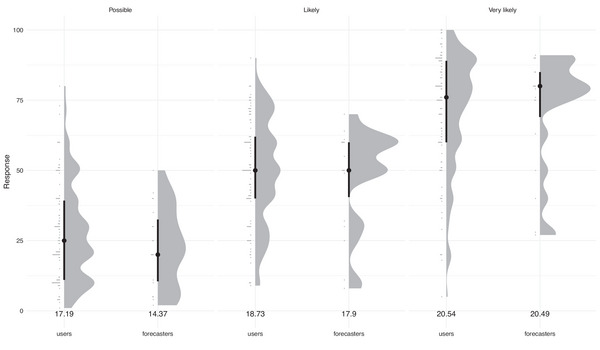
Raincloud plot comparing the responses of professional forecasters and end users when asked to provide a numerical estimate of verbal probability terms (possible, likely, very likely; top of plot) for the frequency response format. For each subplot, the “rain” (horizontal lines to the left) gives the raw data histogram, the “cloud” itself is a kernel density smooth of that data. The median is given by the dot, with the thick line indicating the interquartile range of the data. Numbers under each plot give the standard deviation of that data.

Figure 9 presents professional forecaster estimates including standard deviations alongside end user estimates for the same response format. Mean estimates for “possible,” “likely,” and “very likely” for professional forecasters (22.84, 47.63, 71.79) and end users (27.58, 49.75, 71.51) are broadly similar, though care has to be taken when interpreting these results given the differences in sample size (*N* = 19 vs. *N* = 112). Similarly, we solicited a wide range of estimates from professional forecasters, “possible” ranged from 2 to 50, “likely” from 8 to 64, and “very likely” from 28 to 90.

### 3.3 Discussion

#### 3.3.1 Numerical interpretation of verbal probability terms

We found that end users give significantly different estimates of the term “possible,” “likely,” and “very likely.” However, the estimates are wide‐ranging with large standard deviations associated with each term. Notably, the lowest estimates by end users for “likely” and “very likely” are below 10%, while the highest are 100% (Figure 8).

We found a good match between end users and professional avalanche forecasters considering the mean response, but the variation in the estimates for both forecasters and end users suggests that there is still potential for large discrepancies between individual estimates. Focusing on professional forecasters, who all provided estimates using the frequency format, there was high variability for each verbal probability term. Most notably, 10 out of 19 avalanche forecasters (roughly 53%) interpret “likely” in an avalanche context to be *50% or less* with the lowest estimate for “triggering is likely” meaning 8 out of 100 slopes will avalanche. This interpretation differs sharply from the standard interpretation (Teigen et al., 2022, p. 2).

There might be different reasons for such low estimates by professional forecasters. First, as noted in Section 1, it might be that in the case of avalanches, some forecasters will consider even a 10% probability of triggering an avalanche as a “high” probability, and so using terms like “possible” or even “unlikely” would be inappropriate. In that vein, earlier studies suggest that low base rate contexts with potentially severe outcomes can lower the numerical interpretation of “likely” (Wallsten et al., 1986). Second, the phrase “triggering is likely” is used in the descriptor of a *high* avalanche warning (see Figure 1) and, according to the study by Jamieson et al. (2009), the upper limit for triggering an avalanche at the *high* danger level is 10%. So, awareness of such estimates and knowledge of the relevant detailed descriptors of the danger levels might influence professional forecasters interpretation of verbal probability terms. Finally, as discussed in the next section, the response format may have had an effect on their estimates.

In summary, despite a good match with regard to mean estimates between forecasters and end users, our findings are relevant to avalanche risk communication. The wide range of individual estimates within both user groups for each verbal probability term, coupled with a lack of awareness of this diversity of interpretation, can lead to an “illusion of communication” (Budescu & Wallsten, 1985) between professional forecasters when preparing forecasts, and ultimately to miscommunication when advising the public.

#### 3.3.2 Response formats and variance

In relation to our third aim, this study provides further evidence of the methodological relevance of separating response formats. Consistent with the first study, we found that the response format had an effect on end users' responses and on their variance. Specifically, we found that the frequency format led again to lower average estimates, compared to the other two response formats. Noteworthy, is that we could not identify any differences in estimates between the certainty and percentage chance formats. This suggests that asking for a percentage chance‐estimate or asking for how certain individuals are on a scale from 0 to 100 elicits similar response patterns.

Interestingly, for the frequency format, in contrast to the other two formats, we found that the majority of end users and forecasters (54% and 53%, respectively, compared to 16% and 18% of end users in the certainty and percentage chance formats) interpret “likely” to be 50% or less. This supports the hypothesis that these differences could mainly be a function of the response format. Further research is required to establish whether the frequency format leads to consistently lower estimates in contexts that do not involve rare and severe events, and whether professional forecasters provide different estimates using different response formats.

Finally, we also found that the frequency format led to greater variance than both percentage chance and certainty formats (for the terms “likely” and “very likely”). Assuming that specificity of information is inversely correlated with variance in end user interpretation, the frequency format leads to a possibly more ambiguous interpretation of the verbal probability terms.

In summary, we believe our findings in relation to our third aim strongly suggest that as a *methodological principle*, it is important to control for the effects of different response formats in the context of eliciting numerical estimates of verbal probability terms or other qualitative danger scales, such as the avalanche danger scale.

#### 3.3.3 Limitations of Study 2

There are at least two limitations to highlight: first, we did not specify the relevant avalanche size for which triggering an avalanche is “possible,” “likely,” or “very likely,” and so there is a possibility that individuals had different avalanche sizes in mind when issuing their estimates. Having said this, the Scottish context is one in which large avalanches are exceptionally rare, and so we doubt the size will have much of an effect on their estimates. Still, future studies may want to control for avalanche size when eliciting numerical estimates of trigger likelihood terms.

Second, while we did fix the context in which individuals were asked to issue their estimates and solicited a large number of responses, we did ask respondents to provide single‐valued best estimate responses. However, Budescu and Wallsten (1995) have argued it is beneficial to solicit range estimates, and in future studies, we will adopt a range of different response types.

## 4 GENERAL COMMENTS AND IMPLICATIONS FOR AVALANCHE RISK COMMUNICATION

Our main findings are highly relevant to the practice of avalanche risk communication. Avalanche communicators should do more to highlight the exponential nature of the avalanche danger scale. Failure to understand the exponential nature will ultimately lead to underestimate the relative increase in the probability of triggering an avalanche of higher levels. This is striking, in particular because the *considerable* danger level, which is issued less than 30% of the time, accounts for roughly 50–80% (depending on activity) of fatalities (Winkler et al., 2021).

To address this, avalanche risk communicators may consider using sub‐levels and separate avalanche danger levels into (–, =, +), as adopted by the SLF (see Figure 2). The use of sublevels might facilitate and encourage a better understanding of the exponential nature of the avalanche danger by providing end users with a clearer sense of *where* on the exponential curve the current avalanche danger lies. Of course, the introduction of sublevels requires confidence that these levels can be consistently forecasted.

In relation to the prospects of using numerical estimates in avalanche risk communication, we found that forecasters as well as end users currently adopt a wide range of numerical estimates of the avalanche danger levels and the verbal probability terms. So, should we adopt a more precise numerical presentation mode or better defined verbal probability terms in avalanche risk communication? While the challenges highlighted in Section 1 remain, we have additional doubts motivated by our findings.

We find that respondents *overestimate* the relevant probabilities for triggering an avalanche and *underestimate* the factor by which the risk increases. This discrepancy between the actual and perceived probability of triggering an avalanche brings to the fore a fundamental challenge for *low probability‐high stakes* risk communication:

On the one hand, using standard verbal probability terms, such as “possible,” “likely,” or “very likely” (even including numerical precisifications of these terms), is not well‐suited to capture the trigger probability that individual avalanche decision‐makers *actually* face. These terms have the potential to massively mislead individuals about the personal risks and encourage them to make hugely inaccurate judgments about their personal probability of triggering an avalanche.

On the other hand, using more appropriate verbal probability terms (including their numerical precisifications) is, given the low probability nature of the personal risk, clearly inappropriate and irresponsible: assuming that the probability of triggering on a given slope at avalanche danger level *high* is around 10–20% range, a suitable descriptor would be “unlikely”!

Finally, eschewing with verbal probability terms all together and educating end users about the more realistic numerical estimates of the probability of triggering (especially at the *moderate* level) could quite possibly lead individuals to simply ignore the personal risks. They may well perceive them to be so low that they feel they can simply ignore them.

There are different approaches to resolve the fundamental challenge. Instead of communicating the verbal or numerical probability of triggering an avalanche, forecasters could instead focus on communicating the underlying snowpack sensitivity or the triggering conditions. Forecasters can then use nonprobabilistic notions to communicate the *ease* with which an individual can trigger avalanches using phrases such as “can be triggered,” “easy to trigger,” “very easy to trigger.” While it may be statistically speaking unlikely (10%) for an individual to trigger an avalanche at *high* on a given slope, it strikes us as appropriate to characterize such a situation as one in which it is “very easy” to trigger an avalanche (see Statham et al., 2018 who, however, use different terms). Alternatively, nonprobabilistic notions of risk could be employed to communicate to what extent avalanches are, given the current conditions, to be “expected” or “normal” occurrences—descriptors that are compatible with avalanches being low probability events (Ebert et al., 2020; Kahneman & Tversky, 1982).

Second, forecasters could avoid communicating the absolute risk of triggering an avalanche and instead focus on the increase or decrease of such a risk, that is, the relative risk. For example, communicators could adopt the *moderate* danger level as the baseline avalanche level and communicate other danger levels as increases or decreases of avalanche danger using comparative language such as “more or less likely” and “increased or decreased risk.” Alternatively, forecasters could also use the *low* danger level as the baseline and present each danger level as a significant increase.

We believe repositioning avalanche risk communication by focusing on the relative avalanche risk has a further potential benefit: it is more amenable to the use of numerical estimates. For example, avalanche danger levels (including sublevels) could—provided the data support such judgment—be described as “today the probability of triggering is estimated to be up to 1000 times higher than at *low* avalanche danger” or “the probability of triggering is up to ten times higher today than yesterday.” Hence, a numerical, relative risk strategy is well‐placed to address the fundamental challenge of avalanche risk communication: it encourages end users to make *accurate* (relative) risk judgments, while emphasizing the *exponential* nature of the avalanche danger scale.

## CONFLICT OF INTEREST STATEMENT

The authors declare no potential conflict of interest.

## SUPPORTING INFORMATION

Survey design, data, and R‐code can be downloaded: https://osf.io/3b78y/

## APPENDIX A EFFECT SIZE TABLES

We list the effect sizes for each significance test. Our choice of measure is guided by Tomczak and Tomczak (2014).

**TABLE A1:** Standard deviations and sample sizes per response format and danger level combination.

Question format	Danger level	Standard deviation	*n*
Days	Low	10.023	105
	Moderate	12.84	106
	Considerable	19.063	106
	High	19.47	106
	Very high	18.01	105
Frequency	Low	7.87	107
	Moderate	14.7	106
	Considerable	20.3	106
	High	21.04	106
	Very high	19.63	107
Percentage	Low	10.64	107
	Moderate	14.20	107
	Considerable	19.13	107
	High	20.14	107
	Very high	19.67	107

These are presented in place of effect sizes for Levene's test.

**TABLE A2:** p‐values and effect sizes ($\eta^{2}$ and $E^{2}$) for the Kruskal–Wallis test for differences within the danger levels between response formats.

Danger level	Kruskal–Wallis *p*‐value	$\eta^{2}$	$E^{2}$
Low	2.5$\times 10^{-6}$	0.08	0.08
Moderate	0.01	0.03	0.03
Considerable	0.11	0.01	0.01
High	0.30	0.0	0.01
Very high	0.48	0.0	0.01

Recall $E^{2}$ gives the proportion of the total variance explained by each grouping and $E^{2}$ varies between 0 and 1, indicating no relationship through to full dependence.

**TABLE A3:** Effect sizes and p‐values for the Kruskal–Wallis test between response formats for each verbal probability term.

Verbal probability	Kruskal–Wallis *p*‐value	$\eta^{2}$	$E^{2}$
Possible	$<10^{-16}$	0.11	0.12
Likely	$<10^{-16}$	0.14	0.15
Very likely	$<10^{-16}$	0.10	0.11

Recall $E^{2}$ gives the proportion of the total variance explained by each grouping and $E^{2}$ varies between 0 and 1, indicating no relationship through to full dependence.
